# Our 10-Year Experience with Atrial Myxomas: Is Concurrent Valve Intervention Really Warranted?

**DOI:** 10.21470/1678-9741-2023-0040

**Published:** 2024-01-30

**Authors:** Ketika Potey, Narender Jhajhria, Manish Mallik, Rahul Bhushan, Palash Aiyer, Vijay Grover

**Affiliations:** 1 Department of Cardiothoracic and Vascular Surgery, Dr Ram Manohar Lohia Hospital and Post Graduate Institute of Medical Education and Research, New Delhi, New Delhi, India

**Keywords:** Myxoma, Heart Neoplasms, Echocardiography, Mitral Valve Insufficiency, Dilatation

## Abstract

**Introduction:**

Primary cardiac myxomas are rare tumors. Concurrent valvular lesion is a common finding on evaluation which is thought to be due to annular dilatation secondary to tumor movement across the valve, functional obstruction across the valve, and severe pulmonary hypertension secondary to chronic obstruction. A common belief among surgeons is that excision of myxoma leads to abatement of symptoms, and further valve intervention may not be warranted.

**Methods:**

A 10-year retrospective descriptive study was designed to analyze patients who underwent excision of cardiac myxoma at our center. Data was analyzed regarding presenting features, echocardiographic findings of myxoma and valve morphology, intraoperative assessment, and postoperative outcome with/without valve repair/replacement in all patients.

**Results:**

A total of 22 patients underwent surgery for myxoma. Six patients underwent successful mitral valve repair with ring annuloplasty, two had moderate mitral regurgitation, three had severe mitral regurgitation, and one patient had no mitral regurgitation on preoperative assessment, but moderate mitral regurgitation was found intraoperatively. Four of these patients had no residual mitral regurgitation in follow-up period while two had mild residual mitral regurgitation. One patient had severe mitral stenosis of concurrent rheumatic etiology and successfully underwent mitral valve replacement.

**Conclusion:**

Cardiac myxomas are rare benign tumors commonly associated with mitral valve insufficiency. Mitral valve should be assessed intraoperatively after excision of mass as preoperative assessment might often be insufficient. Concomitant mitral valve intervention might be needed with a case-specific tailored approach, and mitral valve repair with ring annuloplasty offers best surgical outcome in such cases.

## INTRODUCTION

**Table:** 

Abbreviations, Acronyms & Symbols	
AML	= Anterior mitral leaflet
EF	= Ejection fraction
IAS	= Interatrial septum
LA	= Left atrial
LAA	= Left atrial appendage
LV	= Left ventricle
MR	= Mitral regurgitation
MV	= Mitral valve
MVR	= Mitral valve replacement
PML	= Posterior mitral leaflet
RA	= Right atrial
RVSP	= Right ventricular systolic pressure
SJM	= St. Jude Medical
TEE	= Transesophageal echocardiogram
TR	= Tricuspid regurgitation
TV	= Tricuspid valve

Primary cardiac myxomas are rare tumors^[1]^. Myxomas arise from pluripotent mesenchymal stem cells and are most commonly located in the left atrium. Concurrent involvement of mitral valve is a common finding on evaluation which is due to annular dilatation secondary to tumor movement across the valve, functional obstruction across the valve, and severe pulmonary hypertension secondary to chronic obstruction^[2]^. Despite this, excision of myxoma often leads to abatement of symptoms, and further valve intervention may not be warranted^[3]^.

The objective of this study is to retrospectively analyze our center’s specific experience over a period of 10 years in handling cardiac myxoma and the need for concurrent valvular repair/replacement with excision of myxoma. We also aim to correlate preoperative and intraoperative assessment of valve function to establish a correlation between valve disease severity and the intervention needed.

## METHODS

A retrospective descriptive study was designed to analyze patients who underwent excision of cardiac myxoma at our tertiary care center at Atal Bihari Vajpayee Institute of Medical Sciences and Dr Ram Manohar Lohia Hospital (New Delhi, India) from May 2012 to December 2022. A total of 22 patients underwent surgical excision of cardiac myxoma in given time. Data was analyzed regarding presenting features, echocardiographic findings of myxoma and valve morphology, intraoperative assessment, and postoperative outcome with/without valve repair/replacement in all patients.

### Surgical Technique

**Fig. 1:**
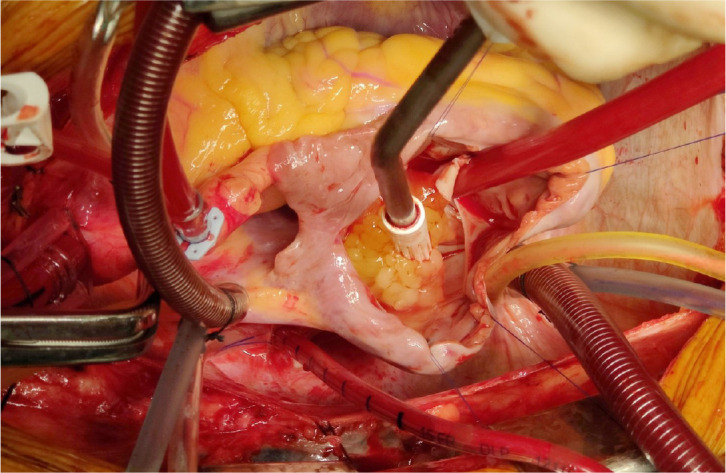
Left atrial myxoma.

All procedures were conducted via median sternotomy and vertical pericardiotomy, with systemic heparinization and total cardiopulmonary bypass with aorto-bicaval cannulation and antegrade cold cardioplegic arrest. Cavae snugged. Myxomas were excised via right or left atriotomy with 5-mm free margin of interatrial septum sent for histopathology (Figure 1). The surgical approach was tailored in accordance with site of attachment of myxoma to interatrial septum or atrial chamber, surgeon’s preference, and size of left atrium. In all cases, mitral valve was evaluated with aid of on-table transesophageal echocardiogram (TEE) and intraoperatively by visual assessment with segmental analysis of leaflets as well as saline insufflation into left ventricle through mitral valve. In six out of 22 patients, a trans left atrial (LA) approach was used, and mitral valve was assessed directly. Eleven patients underwent a bi-atrial repair, and mitral valve was assessed directly in them as well through the LA approach. Five patients underwent a trans right atrial (RA) repair. In these patients, a transseptal approach to mitral valve was used after excision of myxoma. In such patients, if repair of mitral valve was indicated after saline testing, transseptal incision was extended superiorly towards roof of the left atrium while inferiorly it was extended towards inferior vena cavae, and repair was performed. Interatrial septum was closed directly or with a patch. Rewarming was started. Chambers were closed. Cross-clamp was removed. Patient was gradually weaned off cardiopulmonary bypass.

### Follow-up

All patients were routinely followed up - weekly follow-up for six weeks, monthly follow-up for six months, and half-yearly follow-up thereafter.

## RESULTS

A total of 22 patients (Table 1) underwent surgery for myxoma from May 2012 to December 2022.

**Table 1:** Cases’ details.

No	Age (years)/sex	Echocardiography	Surgery performed	Intraoperative finding	MV
1	32/female	Large LA mass moving across MV, moderate TR, EF 60%	Bi-atrial transseptal excision of myxoma	LA myxoma (5 × 5 cm) attached via pedicle to left of IAS	Normal MV
2	48/male	Large LA clot attached to LAA and going to LV in diastole	Trans LA myxoma excision	Myxoma of size 1.5 × 5 cm arising from LAA	Normal MV
3	66/female	4 × 3 cm mass arising from lateral wall of left atrium popping into LV, mild MR	Trans LA myxoma excision	4 × 5 cm myxomatous mass with stalk arising from LAA	Normal MV
4	60/female	Large LA mass attached to IAS of size 3.7 × 2.5 cm, moderate MR, EF 40%	Trans LA myxoma excision	5 × 5 cm gelatinous mass arising from LAA	Normal MV
5	34/female	Large LA mass attached to IAS popping into LV, severe TR, EF 55%, mild MR	Bi-atrial transseptal excision of myxoma	4 × 5 cm LA mass attached to IAS	Normal MV
6	14/female	Large pedunculated mass attached to IAS, low moderate MR, moderate TR	Bi-atrial transseptal excision of myxoma	7 × 5 cm LA mass attached to IAS with pedicle	Normal MV
7	19/female	Large LA mass of 6 × 3.5 cm, severe TR, RVSP 85, severe pulmonary arterial hypertension	Bi-atrial transseptal excision of myxoma with modified De Vega’s TV repair	Large LA mass attached at the level of fossa ovalis, severe TR	Dilated mitral annulus, no concurrent mitral procedure done
8	35/female	Large LA mass of 5 × 4 cm causing mitral obstruction, peak gradient/mean gradient 11/9, severe MR, severe TR	Bi-atrial transseptal excision of myxoma with MV repair and pericardial patch annuloplasty with modified De Vega’s TV repair	5 × 4 cm LA mass attached to IAS, A3 prolapse, dilated mitral and tricuspid annulus	Dilated mitral annulus, MV repair with patch annuloplasty
9	50/female	LA mass of 3 × 5.2 cm protruding into LV, EF 60%	Bi-atrial transseptal excision of myxoma	3 × 6 cm LA mass attached to IAS	Normal MV
10	8/male	Mobile LA mass of 2 × 3.2 cm, moderate MR	Bi-atrial transseptal excision of myxoma with MV repair (SJM Tailor ring #27 annuloplasty)	3 × 3 cm myxoma with broad base attached to base of AML, no septal attachment of myxoma	MV repair (SJM Tailor ring #27 annuloplasty)
11	15/male	Large LA mass of 7 × 5 cm protruding into LV, severe TR	Bi-atrial transseptal excision of myxoma with posteroseptal tricuspid commissuroplasty	7 × 6 cm LA mass attached to IAS, dilated tricuspid annulus	Normal MV
12	52/male	RA mass of 4 × 6.9 cm protruding into right ventricle, EF 50%	Trans RA myxoma excision	Myxomatous mass attached to RA free wall, normal TV	
13	36/male	LA myxoma, severe mitral stenosis, EF 55%	Trans RA myxoma excision and MVR (SJM #31 mitral valve prosthesis)	Lobulated myxoma on LA side, thick AML, diseased MV	MVR (SJM #31 mitral valve prosthesis)
14	5/male	Large LA mass moving across MV, EF 60%	Trans LA myxoma excision	3 × 6 cm LA mass attached to IAS	Normal MV
15	28/female	Mobile LA mass of 4 × 6 cm, moderate MR	Trans RA myxoma excision	4 × 5 cm LA mass attached to IAS with pedicle	Normal MV
16	52/male	LA myxoma, moderate MR, thick AML, restricted PML, mild TR	Trans LA myxoma excision with MV repair (Edwards Physio Ring #30 annuloplasty)	Large LA mass attached to IAS, restricted and thick PML, dilated mitral annulus	Edwards Physio Ring #30 annuloplasty
17	29/female	Large LA mass of 9 × 4.5 cm protruding into LV obstructing MV, EF 50%	Trans RA myxoma excision	9 × 6 cm LA mass attached to IAS	Normal MV
18	21/male	LA mass of 3 × 4 cm protruding into LV, EF 60%	Bi-atrial transseptal excision of myxoma	3 × 4 cm LA mass attached to IAS	Normal MV
19	48/female	Large LA mass of size 6 × 4 cm moving across MV, EF 60%	Bi-atrial transseptal excision of myxoma	6 × 4 cm LA mass attached to IAS	Normal MV
20	27/female	LA myxoma, severe MR, moderate TR, EF 50%	Trans LA myxoma excision with MV repair (Profile 3D™ Ring #30 annuloplasty)	Large LA mass of 5 × 6 cm attached to IAS, restricted and thick PML, dilated mitral annulus	Profile 3D™ Ring #30 annuloplasty
21	50/female	Large LA mass moving across MV, EF 60%	Bi-atrial transseptal excision of myxoma	5 × 4 cm LA mass attached to IAS	Normal MV
22	47/male	Large LA myxoma of 6 × 4 cm, severe eccentric MR, severe TR (RVSP 98)	Trans RA myxoma excision with MV repair (Profile 3D™ Ring #28 annuloplasty) + TV repair (Contour 3D™ ring annuloplasty #28)	6 × 4 cm LA mass attached to IAS	Profile 3D™ Ring #28 annuloplasty

AML=anterior mitral leaflet; EF=ejection fraction; IAS=interatrial septum; LA=left atrial; LAA=left atrial appendage; LV=left ventricle; MR=mitral regurgitation; MV=mitral valve; MVR=mitral valve replacement; PML=posterior mitral leaflet; RA=right atrial; RVSP=right ventricular systolic pressure; SJM=St. Jude Medical; TR=tricuspid regurgitation; TV=tricuspid valve

Median age of the group was 34.5 years. And male to female ratio is 9:13, showing female preponderance for LA myxoma.

**Fig. 2:**
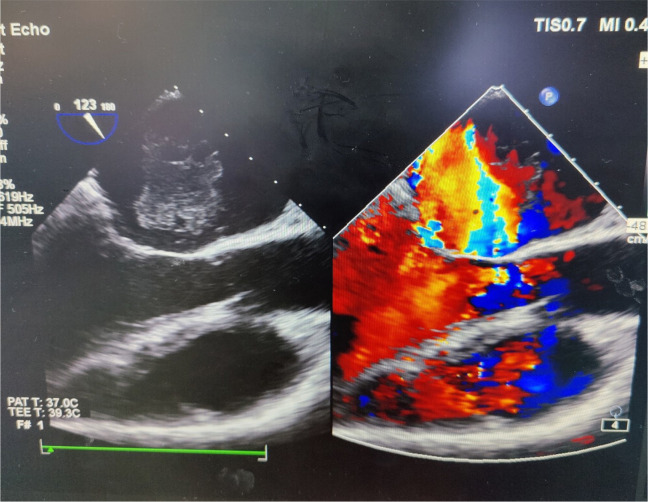
Echocardiogram showing myxoma.

Echocardiographic findings in relation to mitral valve were: six patients were found to have moderate mitral regurgitation (MR), three patients were reported to have severe MR (Figure 2), and one patient was reported to have severe mitral stenosis.

Echocardiographic findings in relation to tricuspid valve were: one patient was reported to have moderate tricuspid regurgitation (TR), and four patients were reported with severe TR.

Trans LA approach was used in six patients while a bi-atrial approach was used in 11 patients. A trans RA approach was used in five patients.

Intraoperatively, out of six patients reported to have moderate MR, three patients (50%) were found to have normal mitral valve leaflet, subvalvular apparatus, and annulus size after excision of myxoma. The findings were confirmed on saline insufflation test and subsequently on intraoperative TEE after weaning off bypass. These patients did not undergo any concurrent valvular intervention. They had no MR on serial postoperative echocardiographic evaluation as well.

**Fig. 3:**
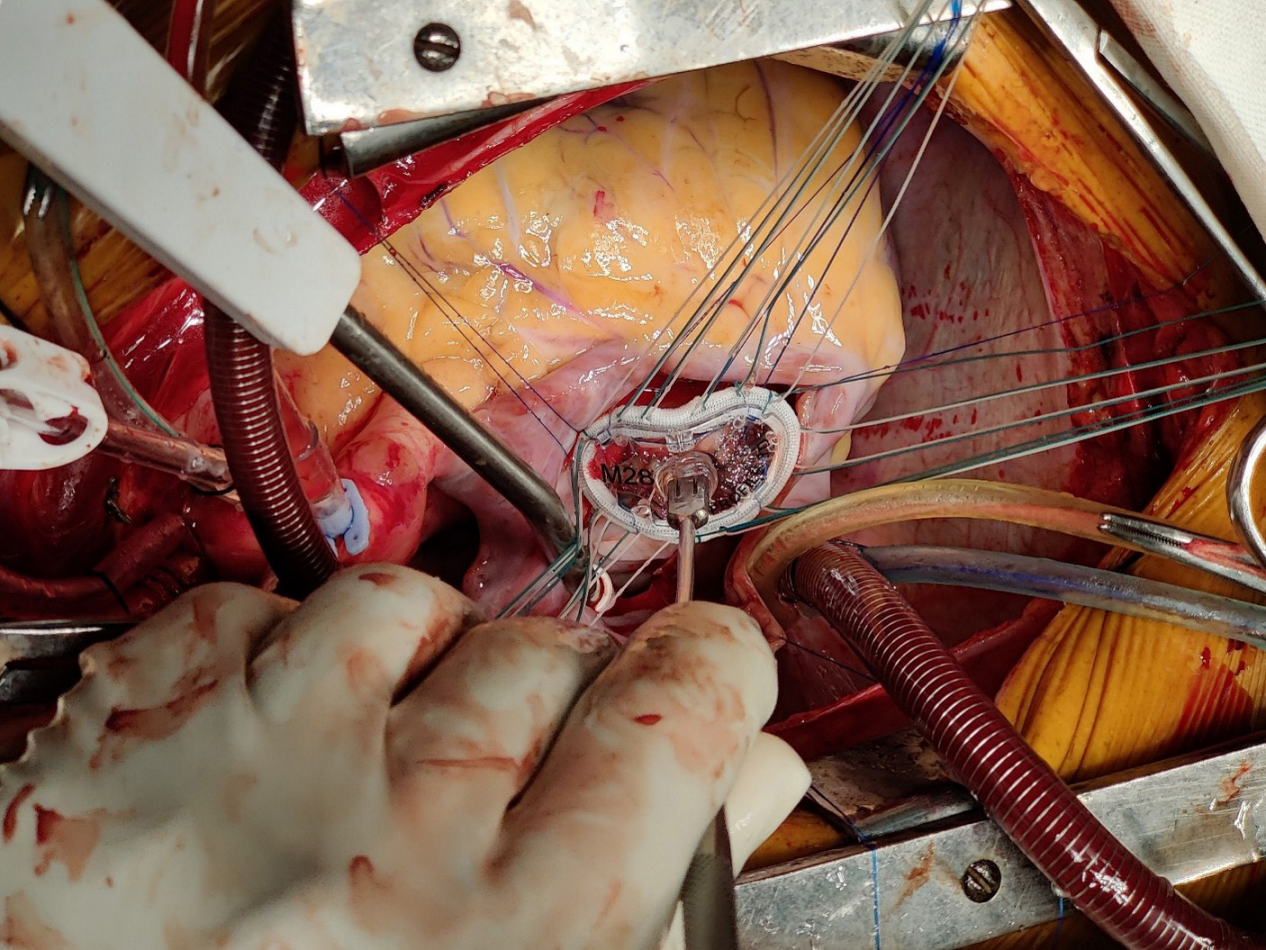
Mitral ring implantation.

The remaining three patients with moderate MR underwent successful mitral valve repair with ring annuloplasty (Figure 3). Two of these patients had no residual MR in follow-up period while one had mild residual MR successfully managed by medical therapy.

Three patients were reported to have severe MR on preoperative echocardiographic evaluation.

Intraoperatively, mitral valve was evaluated with saline insufflation into the left ventricle followed by segmental analysis of anterior and posterior leaflets with a nerve hook. All three patients had dilated mitral valve annulus. Two of these patients had commissural leaks which were managed by commissuroplasty with 5.0 Prolene® sutures. Among them, one patient had additional posterior mitral leaflet (PML) scallop leak which was managed by scallop closure with 5.0 Prolene® suture. Another patient had annular dilatation with prolapsing segment in PML with elongated chordae. This patient was managed by chordal shortening procedure at leaflet level. None of the patients needed leaflet resection or augmentation. All repairs were supported by annuloplasty. Good coaptation with minimal leaks on saline insufflation was accepted, and a confirmational TEE was done intraoperatively after weaning off bypass.

While two patients underwent mitral valve repair with ring annuloplasty, another patient was successfully managed by mitral valve repair with pericardial patch annuloplasty. Two patients had no MR postoperatively, while one patient had residual mild MR on TEE as well as serial postoperative echocardiographic monitoring, and they were managed successfully on medical therapy.

One patient reported to have severe mitral stenosis was found to have thick bulging and calcified leaflets and tethered chordae suggestive of concurrent rheumatic etiology. This patient successfully underwent a mitral valve replacement with St. Jude Medical #31 sized valve.

While four patients were reported to have moderate TR, other four patients were reported to have severe TR. There was no concurrent tricuspid valve intervention needed in patients with moderate secondary TR. Out of four patients with severe TR, a successful De Vega’s repair was carried in two of these patients, ring annuloplasty was carried out in one patient, while a posteroseptal commissuroplasty was carried out in one patient to control TR. In the postoperative period, while three patients had mild TR in follow-up, one patient had moderate TR, which was managed successfully by fluid restriction and diuretics and needed no further surgical intervention.

## DISCUSSION

Myxomas are the most prevalent primary cardiac tumors, with 80-85% found in the left atrium^[4]^. The common age group for cardiac myxoma is the third to fifth decade of life. Female sex is more commonly affected by this disease subset as evident by findings of our study as well^[5,6]^. The tendency of MR and prolapse is common in LA myxomas due to pendulum-like motion of myxomatous mass across mitral valve in each cardiac cycle leading to annular dilatation and leaflet prolapse^[7]^. This can also lead to complications such as endocarditis, atrial fibrillation, thromboembolism, and pulmonary hypertension, thus warranting a timely intervention^[8]^. An extensive preoperative and intraoperative mitral valve assessment is needed to evaluate valve dynamics, geometry, and valvular and subvalvular apparatus to classify functional MR^[9,10]^.

A bi-atrial surgical approach is helpful to determine the correct resection margin by confirming the tumor pedicle under direct visualization, to minimize handling of the tumor, through evaluation of all heart chambers and to evaluate mitral valve completely^[11]^.

As evident from our study, 50% of the patients reported to have moderate MR did not need any concurrent valvular intervention after excision of myxoma. These patients did not have any residual MR as well in follow-up period. The remaining 50% of patients were successfully managed by mitral valve repair with ring annuloplasty. In those patients diagnosed with severe MR (three patients), a successful mitral valve repair with ring annuloplasty/pericardial patch annuloplasty was carried out, thus obviating the need for valve replacement. Valve replacement was carried out in a single patient with stenotic mitral valve with rheumatic etiology.

In one of the first case reports of LA myxoma causing mitral incompetence, Blanco et al. managed to repair PML and performed an annuloplasty to limit postoperative mitral incompetence^[12]^. Myxomas are a rare entity, and myxomas associated with valvular incompetence are further rarer; there lies a lack of detailed studies and evidences for recommendations for concurrent valvular intervention in such cases^[13]^. In the landmark study published by Lee et al.^[1]^ discussing their 30-year experience with atrial myxomas, 5.3% (five out of 93) of patients operated for myxomas needed concurrent mitral valve intervention. While a valve replacement was carried out primarily in three out of five patients, two patients were managed by valve repair in their study.

The limited literature on cardiac myxoma advises for tailored approach for each individual patient reported to have concurrent mitral valve intervention. While moderate MR might not need any intervention after excision of myxomatous mass, an intervention of mitral repair is almost always a complete solution thus obviating a need for surgical valve replacement^[14]^. Surgeons’ tailored approach with careful native valve preservation shall give optimum valvular incompetence relief to patient^[15]^.

### Limitations

The limitations of our study are it being a retrospective analysis and the limited number of patients owing to rarity of this disease.

## CONCLUSION

Cardiac myxomas are rare benign tumors commonly associated with mitral valve insufficiency. A thorough assessment of the mitral valve intraoperatively, with respect to valve morphology and subvalvular apparatus, after excision of mass is imperative as preoperative assessment might often be insufficient. Concomitant mitral valve intervention might be needed in case-specific tailored approach, and various mitral valve repair techniques with a ring annuloplasty can be implemented to achieve optimal surgical outcome.

**Table:** 

Authors’ Roles & Responsibilities	
KP	Substantial contributions to the conception of the work; and the acquisition of data for the work; final approval of the version to be published
NJ	Revising the work; final approval of the version to be published
MM	Substantial contributions to the acquisition of data for the work; final approval of the version to be published
RB	Substantial contributions to the conception of the work; drafting the work; final approval of the version to be published
PA	Revising the work; final approval of the version to be published
VG	Revising the work; final approval of the version to be published
